# Supplementary figures and images for: Structure of human glycoprotein 2 reveals mechanisms underlying filament formation and adaption to proteolytic environment in the digestive tract
Source: PLoS Biol. 2025 Jun 23;23(6):e3003238. doi: 10.1371/journal.pbio.3003238 (PMC12212870; doi:10.1371/journal.pbio.3003238)

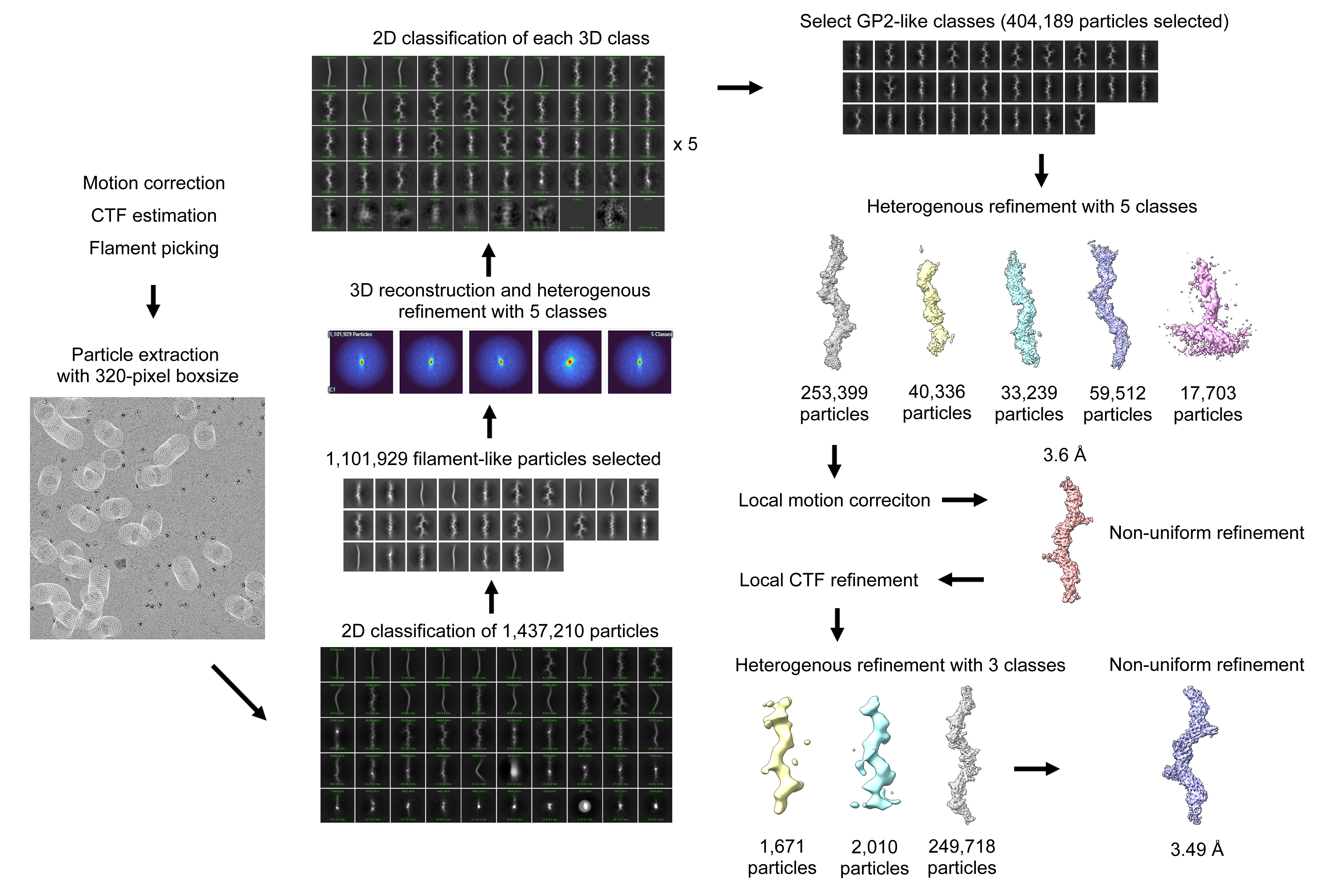

Supplement: S1 Fig — (TIF) [file pbio.3003238.s001.tif]

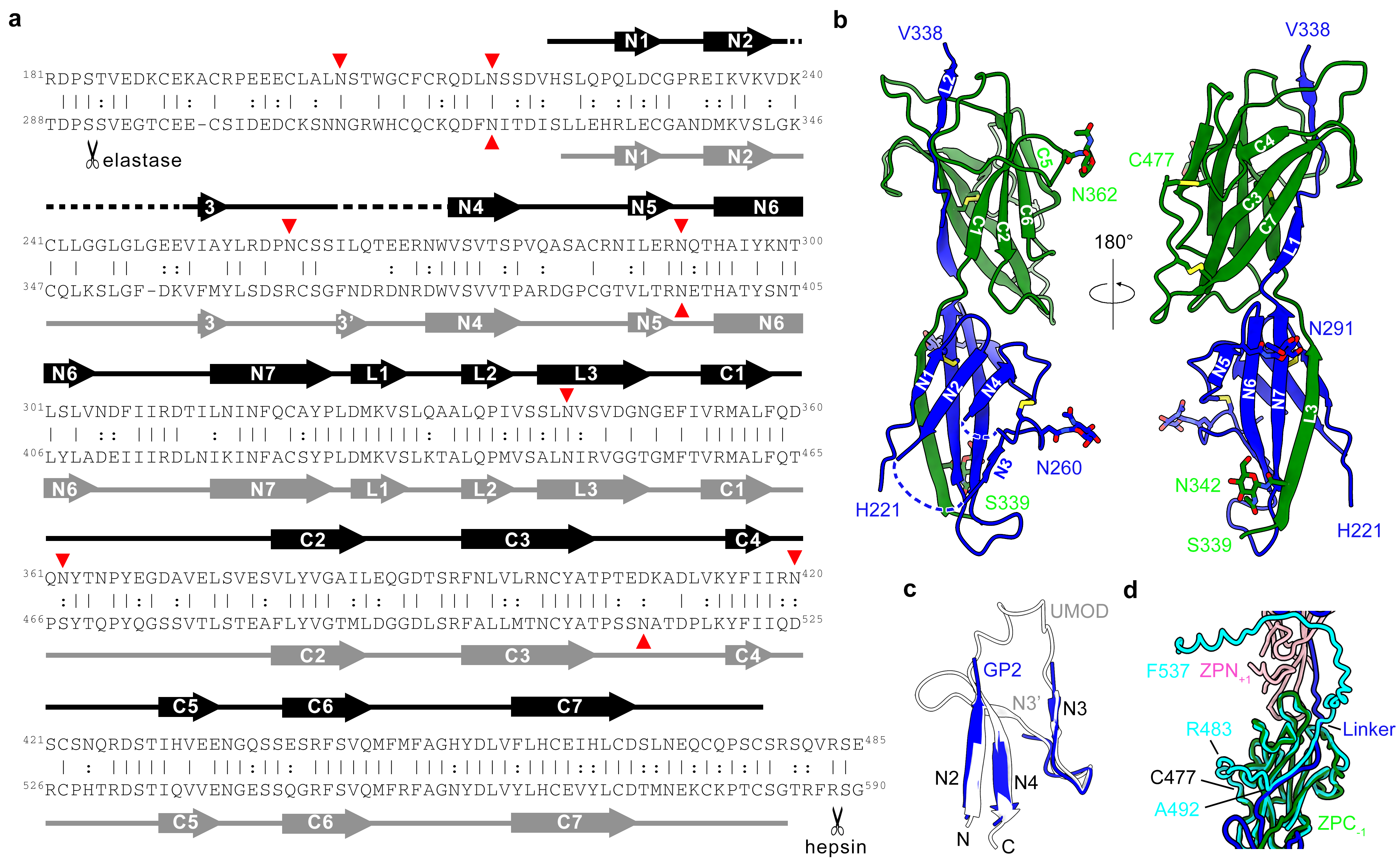

Supplement: S3 Fig — a, Sequence alignment of human GP2 (top) and UMOD (bottom). Secondary structures of each protein are aligned with their amino acid sequences, shown in black for GP2 and grey for UMOD. β-strands are indicated with arrows, loops with solid lines, and flexible loops with dashed lines. N-glycosylation sites observed on filament structures are marked with red triangles, and the elastase and hepsin cleavage sites of UMOD are indicated with scissor marks. b, Cryo-EM structure of GP2 filament, with β-strands labeled. c, Structure superimposition of GP2 and UMOD in the flexible loop region. d, structural alignment of cryo-EM structure of GP2 filament and the AlphaFold model of full length GP2, the linker from a given ZP module is colored in blue and the ZPN and ZPC from the previous and subsequent ZP modules are colored in pink (ZPN+1) and green (ZPC−1), respectively. The AlphaFold model is colored in cyan. (TIF) [file pbio.3003238.s003.tif]

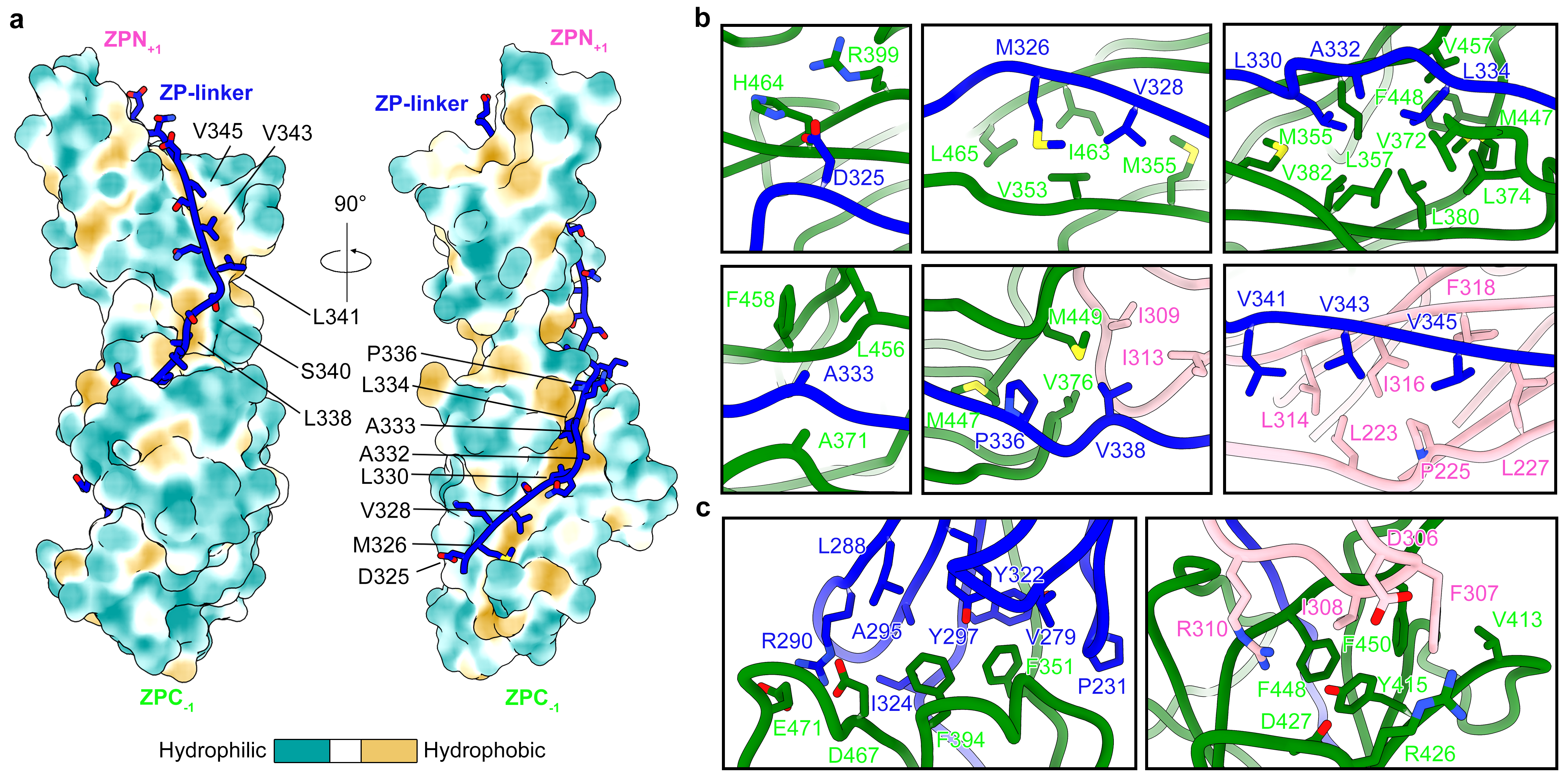

Supplement: S4 Fig — a–b, Interactions between the ZP-linker (blue) and the preceding ZPC domain (ZPC−1, green) and the subsequent ZPN domain (ZPN+1, pink) in general views (a) and detailed views (b). In panel a, ZPN+1 and ZPC−1 are represented as surfaces, colored based on the molecular lipophilicity potential, ranging from most hydrophilic (dark cyan) to most hydrophobic (dark goldenrod). c, Detailed interactions between ZPN (blue) and ZPC−1 (green, left panel) and between ZPC−1 (green) and ZPN+1 (pink, right panel). The electrostatic interactions shown in panels b and c involve residues Asp325, His464 and Arg399 (panel b, top left), Arg290, Asp467, and Glu471 (panel c, left), Arg310 and Asp427 (panel c, right), and Asp306 and Arg426 (panel c, right). (TIF) [file pbio.3003238.s004.tif]

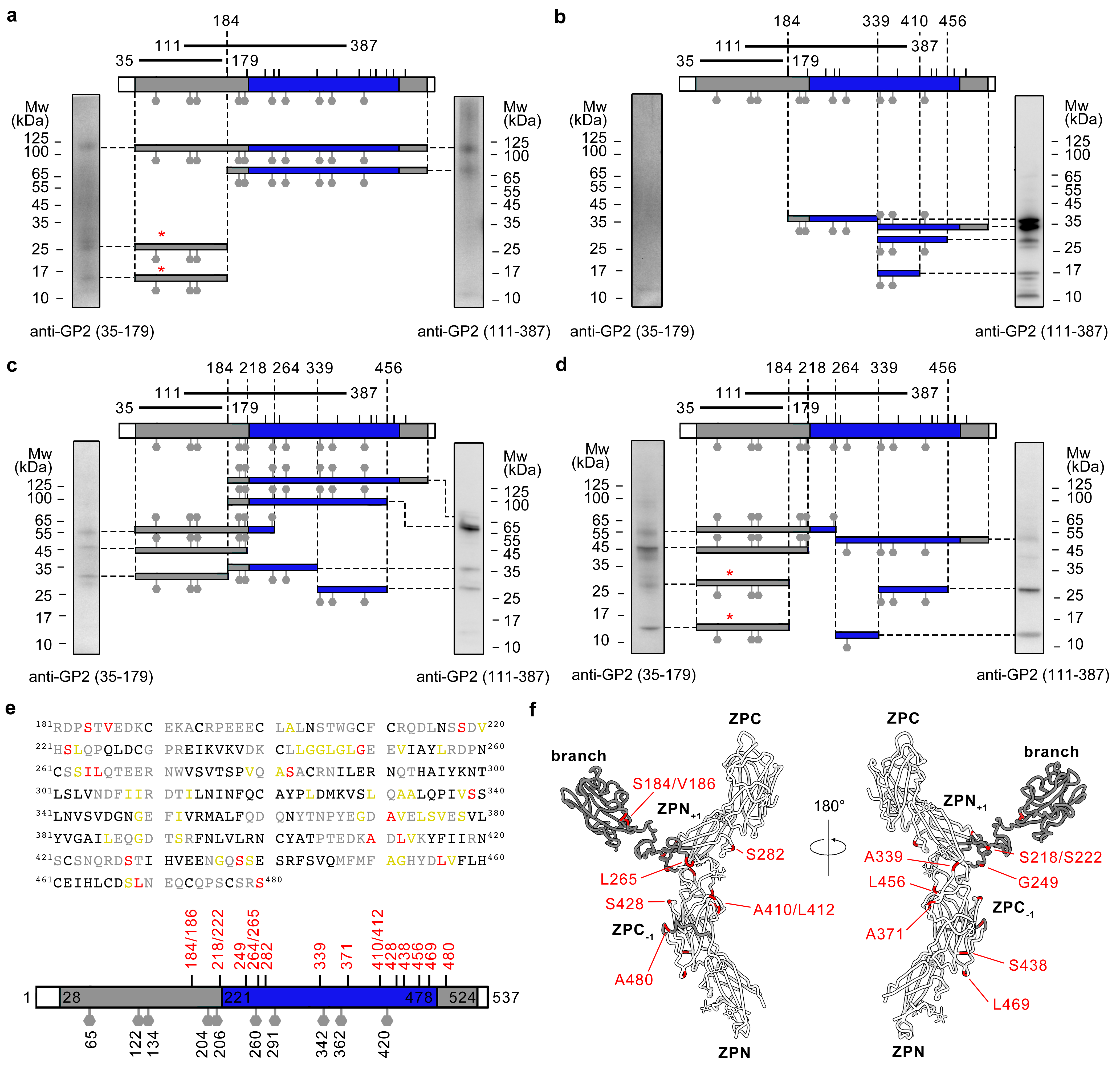

Supplement: S5 Fig — a–d, Residue range predictions of each band in (a) pancreas-derived GP2 without elastase cleavage (first lane in Fig 3a), (b) pancreas-derived GP2 with elastase cleavage (second lane in Fig 3a), (c) small-intestine-derived GP2 from donor 2 (fourth lane in Fig 3a), or (d) donor 3 (fifth lane in Fig 3a). The contrast of the western blotting has been manually adjusted for better presentation of the bands, and the unadjusted blots are shown in Fig 3a and in S1 Raw Images. Since the band patterns in donor 4 are similar to those of donor 3, we used donor 3 to represent both donors. For detailed analysis, please refer to S1 Supplementary Notes (Notes 1–3) and S2 Table. e, The upper panel displays the amino acid sequence of GP2, 181–480, with color coding as described in S1 Supplementary Notes (Note 2). The lower panel shows a schematic of GP2, with N-glycosylation sites labeled and colored in grey, and potential elastase cleavage sites labeled and colored in red. f, The structure of GP2 filament with potential elastase cleavage sites labeled and colored in red. The cryo-EM model is colored in white, and the missing parts are complemented by the alpha-fold model, colored in grey. (TIF) [file pbio.3003238.s005.tif]

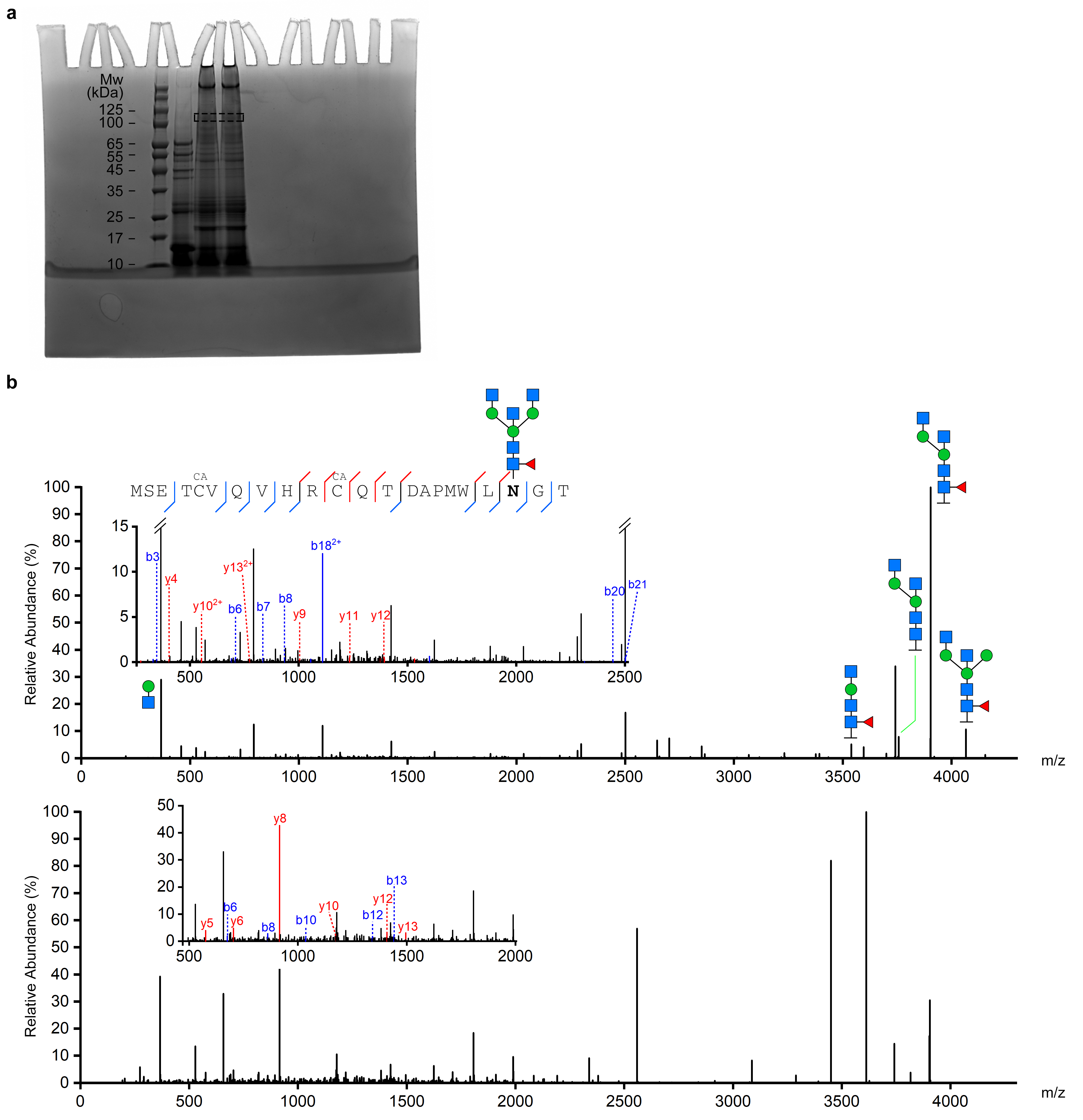

Supplement: S7 Fig — a, SDS–PAGE gel of GP2 filaments extracted from donor 1. The dashed box indicates the gel region corresponding to the approximately 105 kDa band from the GP2 western blot (Fig 3a), which was excised for LC–MS/MS analyses. The original gel can be found in S1 Raw Images. b, (Upper panel) LC–MS/MS spectra of the GP2 glycopeptide containing Asn122, with its sequence shown in the top left corners. Detected peptide-backbone fragment ions are labeled as red and blue lines. (Lower panel) LC–MS/MS spectra of the glycopeptide detected using GP2-free database (from a glycoprotein other than GP2), which exhibited a similar mass to the GP2 glycopeptide shown in the upper panel. CA, carbamidomethylation. N-glycan structures are depicted following the Consortium for Functional Glycomics (CFG) notation: blue square, N-acetylglucosamine; green circle, mannose; yellow circle, galactose; red triangle, fucose. (The legend also applies to S8–S15 Figs). (TIF) [file pbio.3003238.s007.tif]

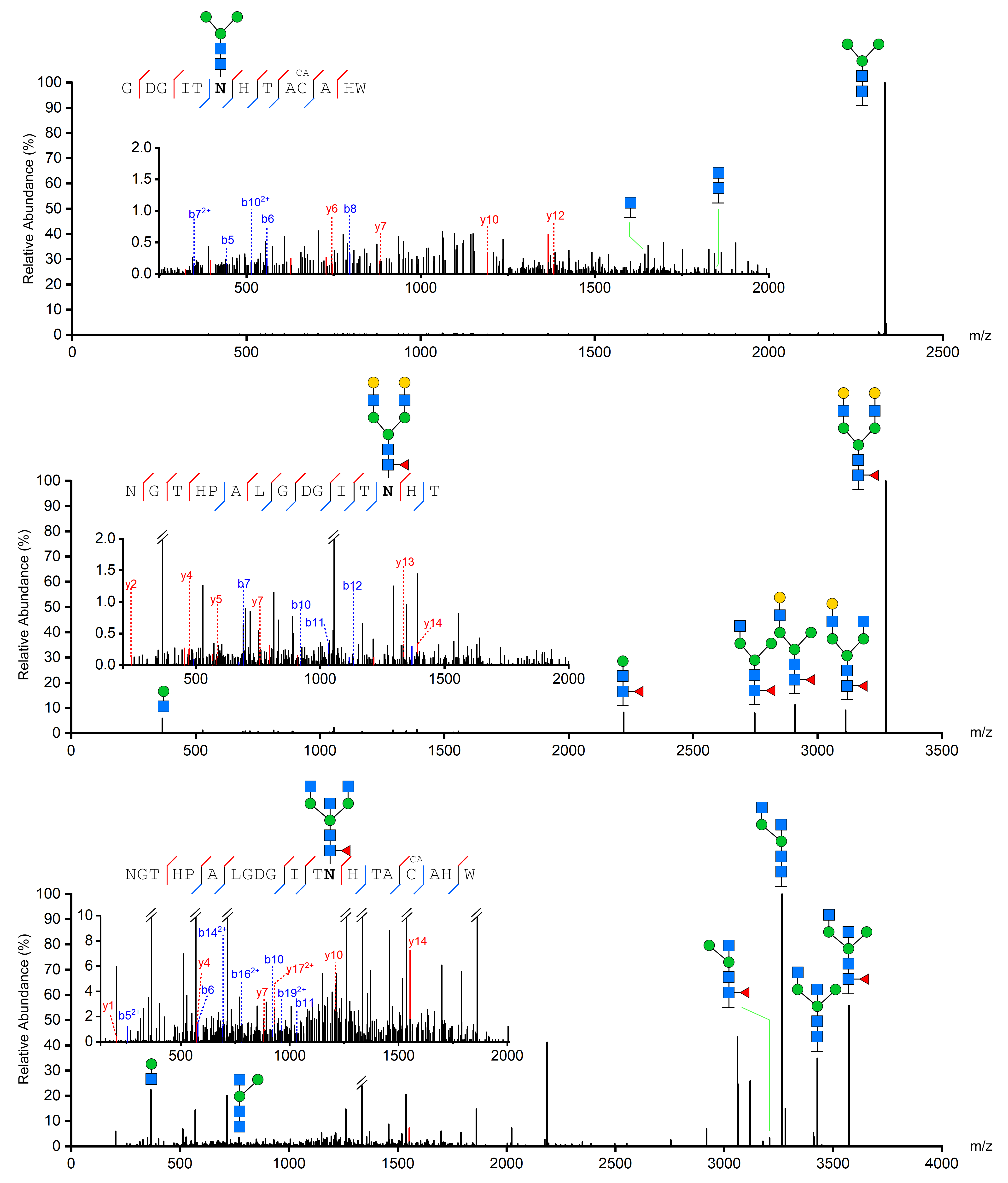

Supplement: S8 Fig — (TIF) [file pbio.3003238.s008.tif]

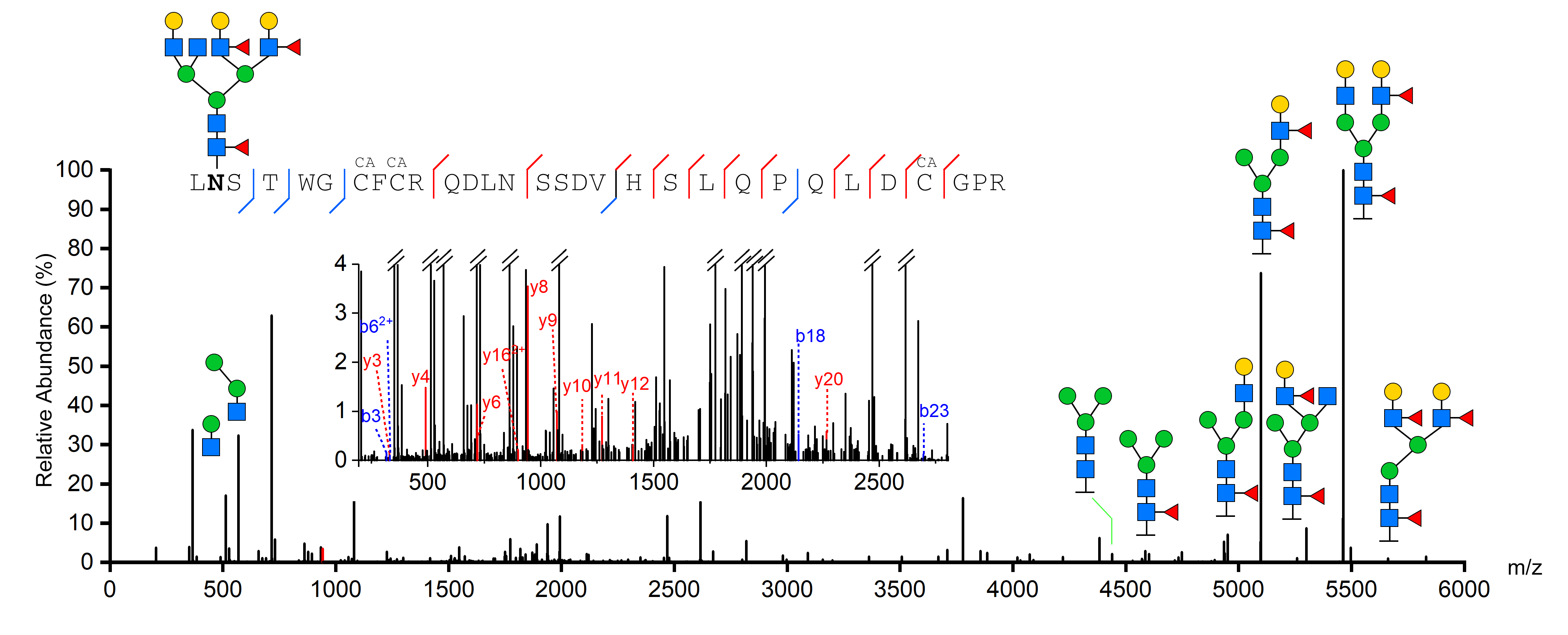

Supplement: S9 Fig — (TIF) [file pbio.3003238.s009.tif]

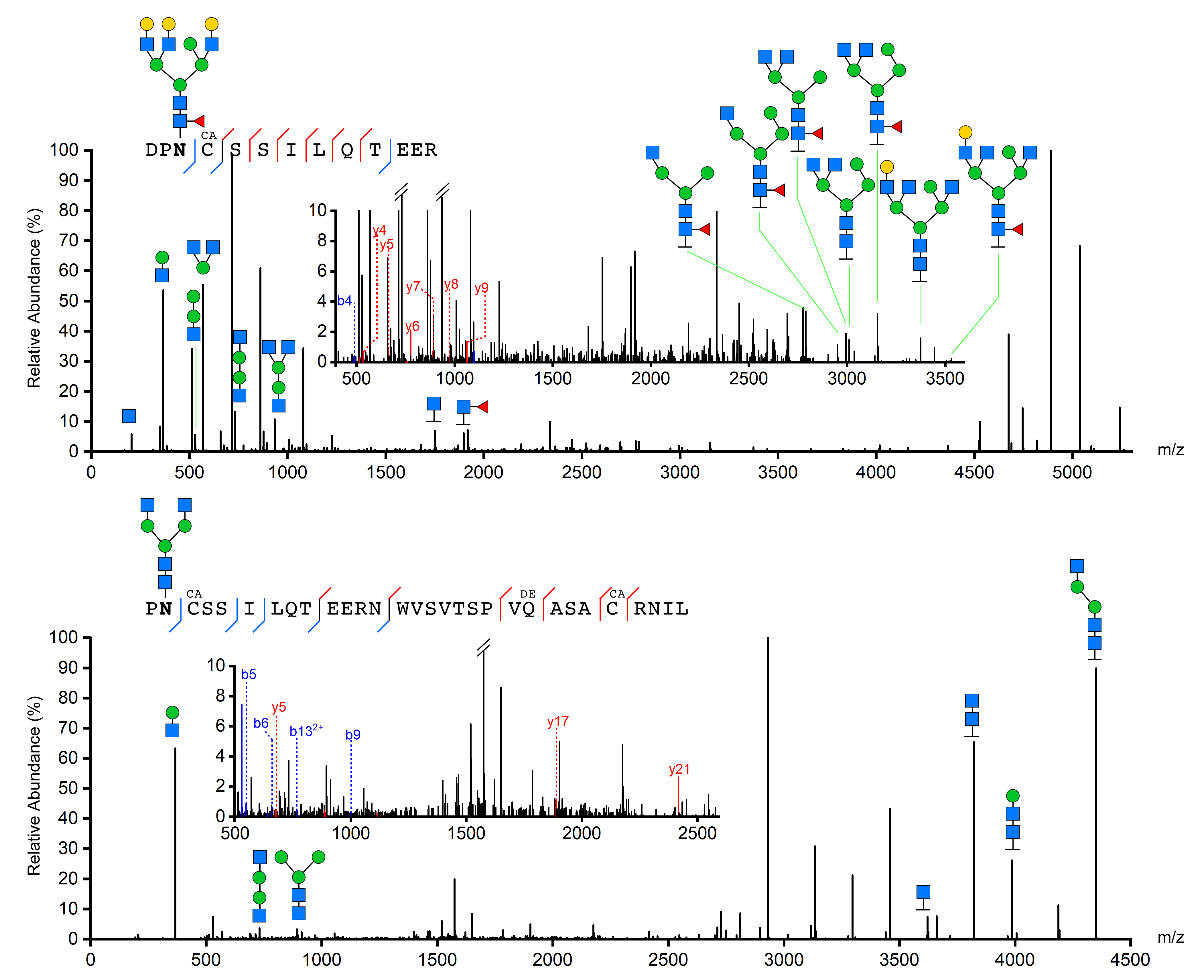

Supplement: S11 Fig — (TIF) [file pbio.3003238.s011.tif]

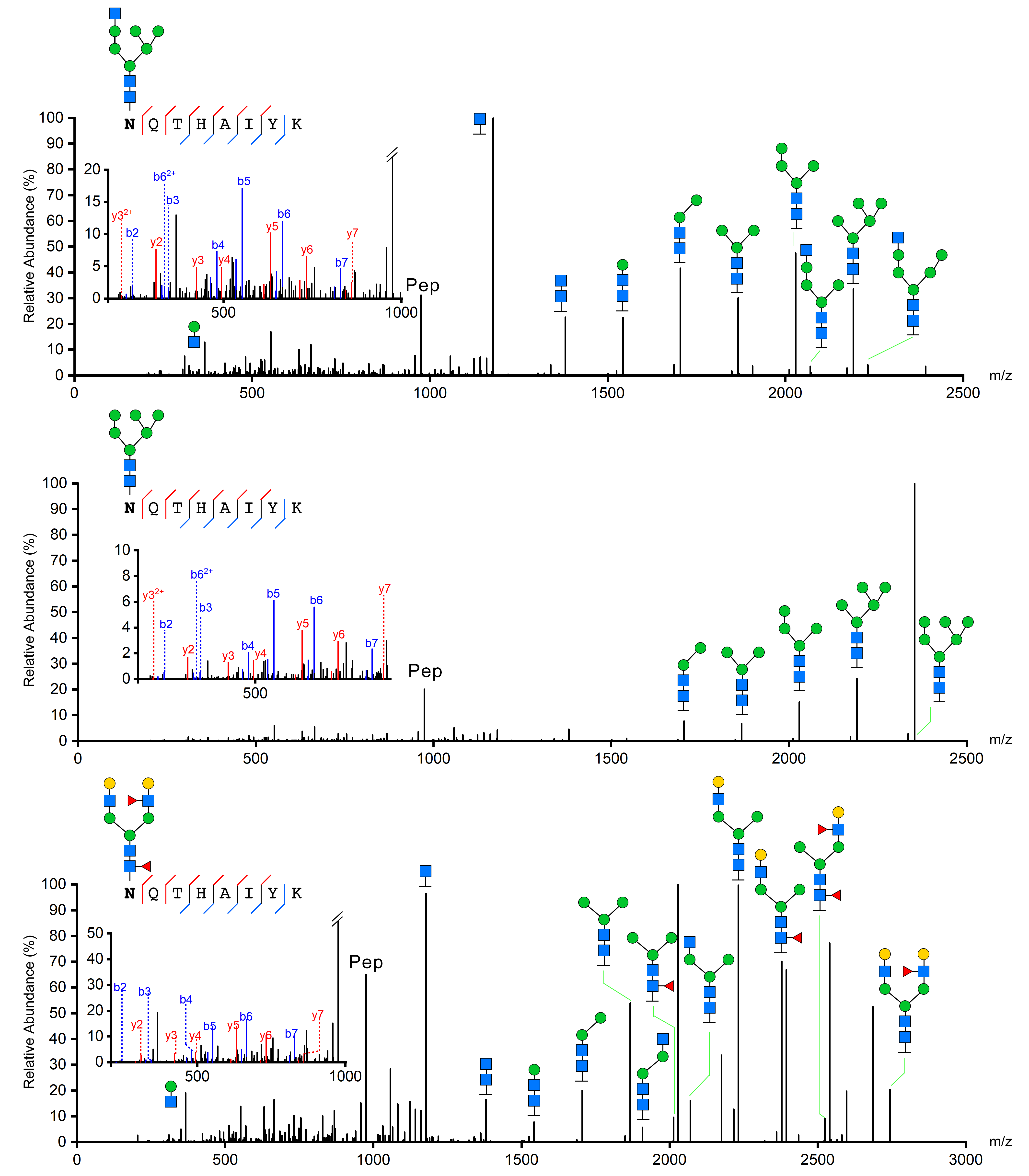

Supplement: S12 Fig — (TIF) [file pbio.3003238.s012.tif]

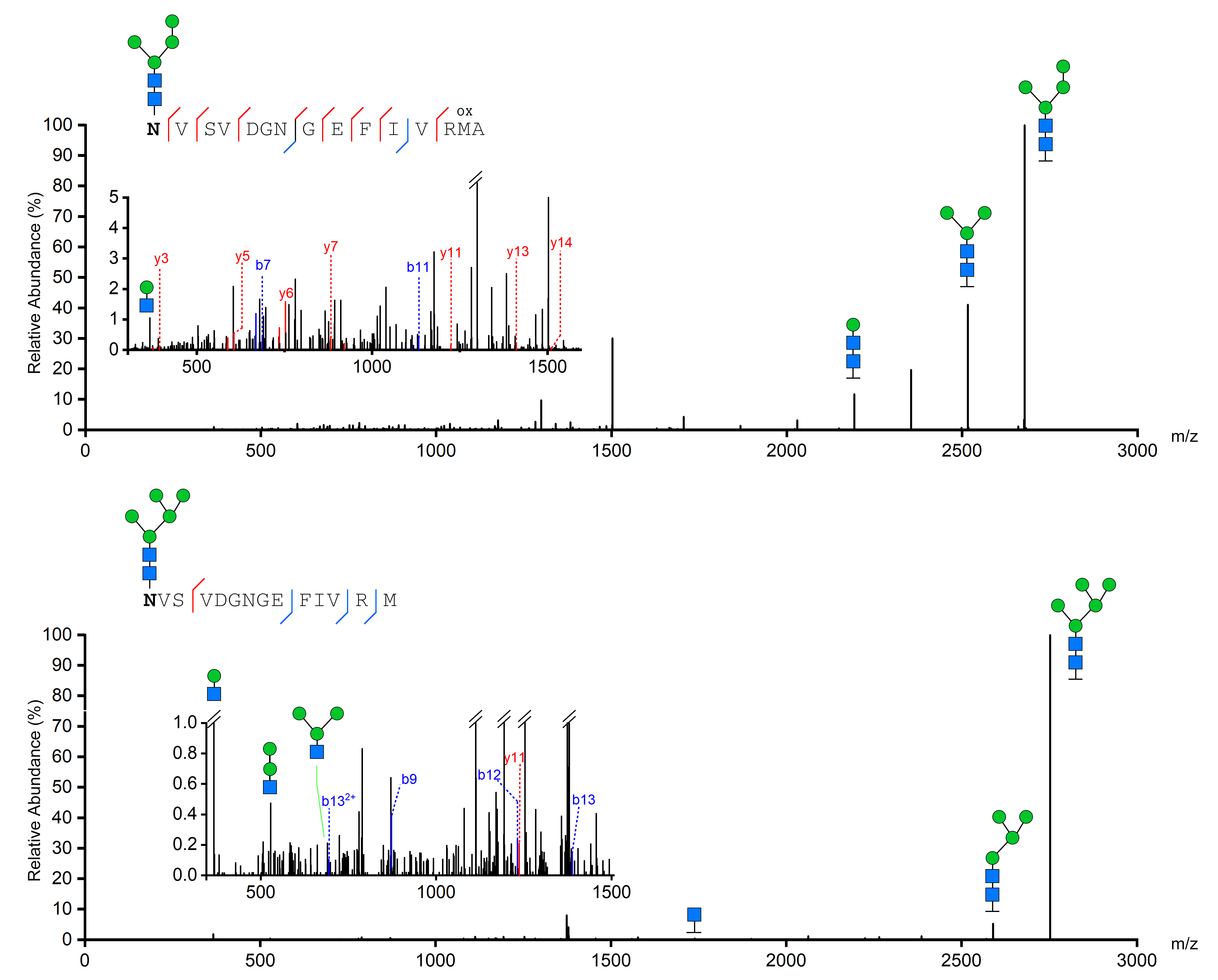

Supplement: S13 Fig — OX, oxidation. (TIF) [file pbio.3003238.s013.tif]

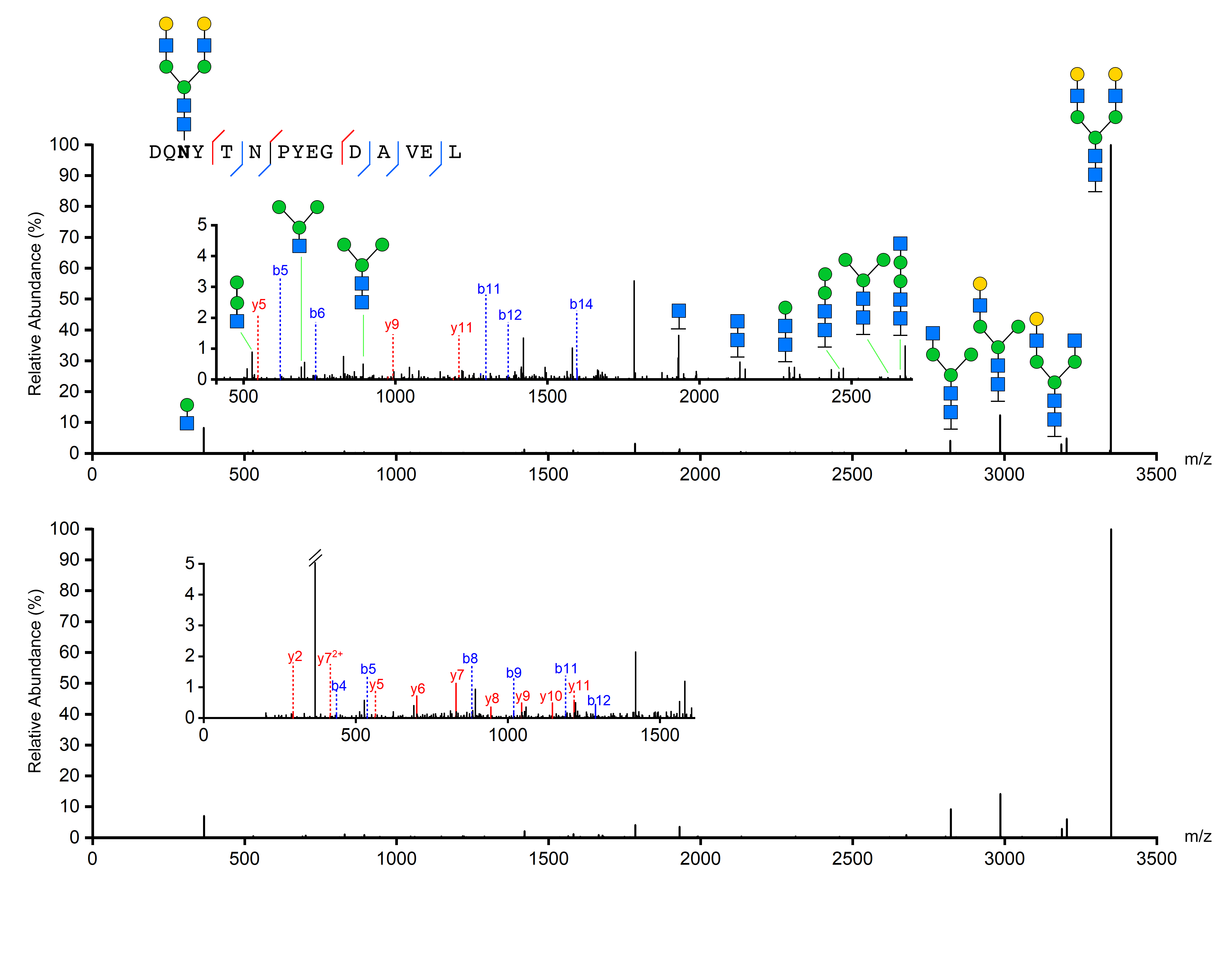

Supplement: S14 Fig — The lower panel displays LC–MS/MS spectra of the glycopeptide detected using GP2-free database (from a glycoprotein other than GP2), which exhibited a similar mass to the GP2 glycopeptide shown in the upper panel. (TIF) [file pbio.3003238.s014.tif]

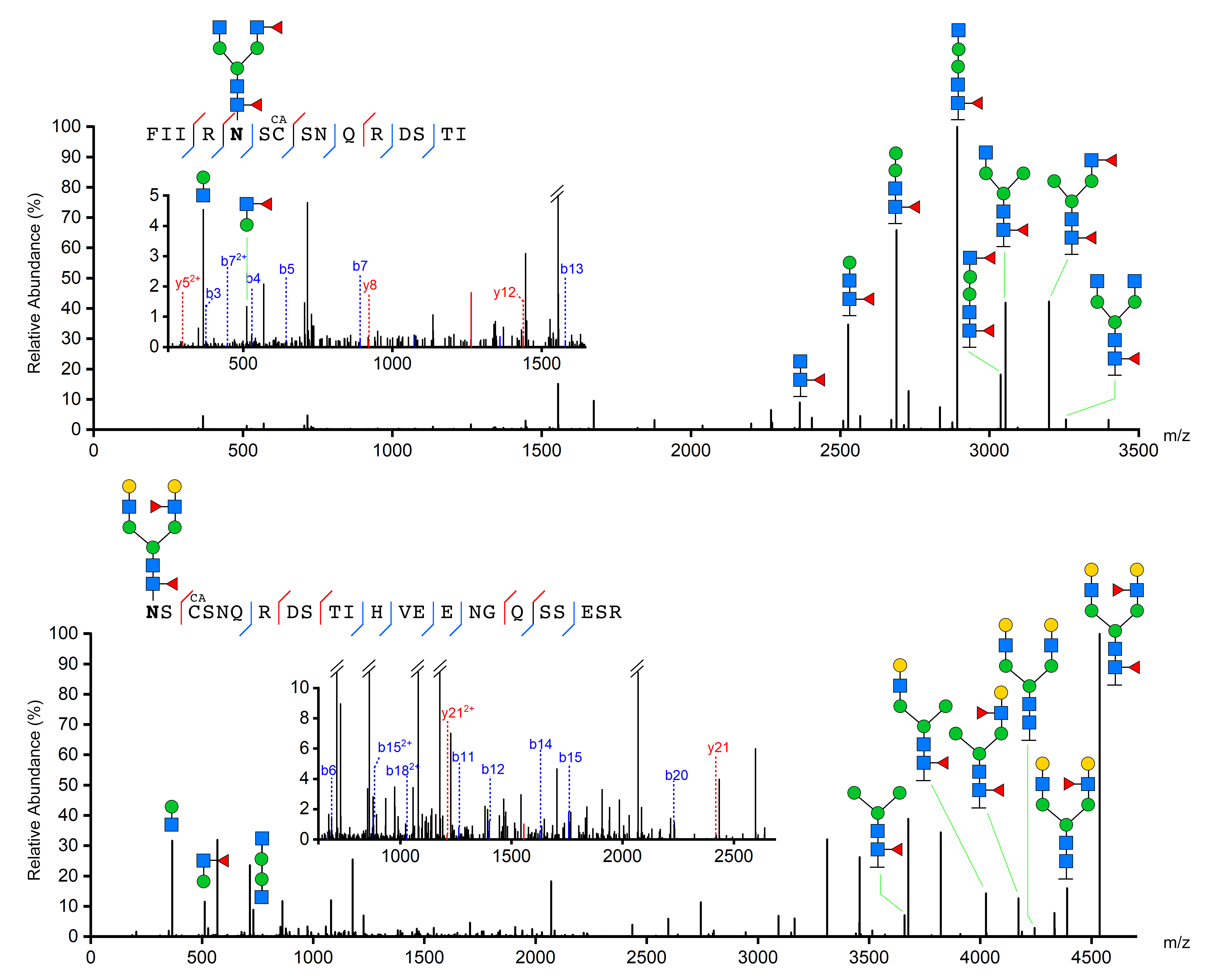

Supplement: S15 Fig — (TIF) [file pbio.3003238.s015.tif]
