# Supplementary material for: Structure of human glycoprotein 2 reveals mechanisms underlying filament formation and adaption to proteolytic environment in the digestive tract
Source: PLoS Biol. 2025 Jun 23;23(6):e3003238. doi: 10.1371/journal.pbio.3003238 (PMC12212870; doi:10.1371/journal.pbio.3003238)
Supplement: S1 Table — (PDF) [file pbio.3003238.s016.pdf]

S1 Table Primers used for GP2 genotyping and the genotyping results

Primers	Sequences (5’-3’)
gp2_1-forward	ATGCCTCACCTTATGGAAAGGATGGTGGG
gp2_1-reverse	ACCCAGTTCCTCTCCTCTGTCTGCAAG
gp2_2-forward	ATCTGTGACCAGCCCCGTCCAG
gp2_2-reverse	TCAGAACAGCCAAGCCAGGAGGACAG
Genotyping	Sequences (5’-3’)
Isoform 3	ATGCCTCACCTTATGGAAAGGATGGTGGGCTCTGGCCTCCTGTGGCTGGCCTTG<br>GTCTCCTGCATTCTGACCCAGGCATCTGCAGTGCAGCGAGGTTATGGAAACCCC<br>ATTGAAGCCAGTTCGTATGGGCTGGACCTGGACTGCGGAGCTCCTGGCACCCCA<br>GAGGCTCATGTCTGTTTTGACCCCTGTCAGAATTACACCCTCCTGGATGAACCCT<br>TCCGAAGCACAGAGAACTCAGCAGGGTCCCAGGGGTGCGATAAAAACATGAGC<br>GGCTGGTACCGCTTTGTAGGGGAAGGAGGAGTAAGGATGTCGGAGACCTGTGT<br>CCAGGTGCACCGATGCCAGACAGACGCTCCCATGTGGCTGAATGGGACCCACC<br>CTGCCCTTGGGGATGGCATCACCAACCACACTGCCTGTGCCCATTGGAGTGGCA<br>ACTGCTGTTTCTGGAAAACAGAGGTGCTGGTGAAGGCCTGCCCAGGCGGGTACC<br>ATGTGTACCGGTTGGAAGGCACTCCCTGGTGTAATCTGAGATACTGCACAGACC<br>CATCCACTGTGGAGGACAAGTGTGAGAAGGCCTGCCGCCCCGAGGAGGAGTGC<br>CTTGCCCTCAACAGCACCTGGGGCTGTTTCTGCAGACAGGACCTCAATAGTTCT<br>GATGTCCACAGTTTGCAGCCTCAGCTAGACTGTGGGCCCAGGGAGATCAAGGTG<br>AAGGTGGACAAATGTTTGCTGGGAGGCCTGGGTTTGGGGGAGGAGGTCATTGC<br>CTACCTGCGAGACCCAAACTGCAGCAGCATCTTGCAGACAGAGGAGAGGAACT<br>GGGTATCTGTGACCAGCCCCGTCCAGGCTAGTGCCTGCAGGAACATTCTGGAGA<br>GAAATCAAACCCATGCCATCTACAAAAACACCCTCTCCTTGGTCAATGATTTCA<br>TCATCAGAGACACCATCCTCAACATCAACTTCCAATGTGCCTACCCACTGGACA<br>TGAAAGTCAGCCTCCAAGCTGCCTTGCAGCCCATTGTAAGTTCCCTGAACGTCA<br>GTGTGGACGGGAATGGAGAGTTCATTGTCAGGATGGCCCTCTTCCAAGACCAGA<br>ACTACACGAATCCTTACGAAGGGGATGCAGTTGAACTGTCTGTTGAGTCCGTGC<br>TGTATGTGGGTGCCATCTTGGAACAAGGGGACACCTCCCGGTTTAACCTGGTGT<br>TGAGGAACTGCTATGCCACCCCCACTGAAGACAAGGCTGACCTTGTGAAGTATT<br>TCATCATCAGAAACAGCTGCTCAAATCAACGTGATTCCACCATCCACGTGGAGG<br>AGAATGGGCAGTCCTCGGAAAGCCGGTTCTCAGTTCAGATGTTCATGTTTGCTG<br>GACATTATGACCTAGTTTTCCTGCATTGTGAGATTCATCTCTGTGATTCTCTTAA<br>TGAACAGTGCCAGCCTTCTTGCTCAAGAAGTCAAGTCCGCAGTGAAGTACCGGC<br>CATCGACCTAGCCCGGGTTCTAGATTTGGGGCCCATCACTCGGAGAGGTGCACA<br>GTCTCCCGGTGTCATGAATGGAACCCCTAGCACTGCAGGGTTCCTGGTGGCCTG<br>GCCTATGGTCCTCCTGACTGTCCTCCTGGCTTGGCTGTTCTGA
Isoform 4	ATGCCTCACCTTATGGAAAGGATGGTGGGCTCTGGCCTCCTGTGGCTGGCCTTG<br>GTCTCCTGCATTCTGACCCAGGCATCTGCAGTGCAGCGAGACCCATCCACTGTG<br>GAGGACAAGTGTGAGAAGGCCTGCCGCCCCGAGGAGGAGTGCCTTGCCCTCAA<br>CAGCACCTGGGGCTGTTTCTGCAGACAGGACCTCAATAGTTCTGATGTCCACAG<br>TTTGCAGCCTCAGCTAGACTGTGGGCCCAGGGAGATCAAGGTGAAGGTGGACA

	AATGTTTGCTGGGAGGCCTGGGTTTGGGGGAGGAGGTCATTGCCTACCTGCGAG<br>ACCCAAACTGCAGCAGCATCTTGCAGACAGAGGAGAGGAACTGGGTATCTGTG<br>ACCAGCCCCGTCCAGGCTAGTGCCTGCAGGAACATTCTGGAGAGAAATCAAAC<br>CCATGCCATCTACAAAAACACCCTCTCCTTGGTCAATGATTTCATCATCAGAGA<br>CACCATCCTCAACATCAACTTCCAATGTGCCTACCCACTGGACATGAAAGTCAG<br>CCTCCAAGCTGCCTTGCAGCCCATTGTAAGTTCCCTGAACGTCAGTGTGGACGG<br>GAATGGAGAGTTCATTGTCAGGATGGCCCTCTTCCAAGACCAGAACTACACGAA<br>TCCTTACGAAGGGGATGCAGTTGAACTGTCTGTTGAGTCCGTGCTGTATGTGGG<br>TGCCATCTTGGAACAAGGGGACACCTCCCGGTTTAACCTGGTGTTGAGGAACTG<br>CTATGCCACCCCCACTGAAGACAAGGCTGACCTTGTGAAGTATTTCATCATCAG<br>AAACAGCTGCTCAAATCAACGTGATTCCACCATCCACGTGGAGGAGAATGGGC<br>AGTCCTCGGAAAGCCGGTTCTCAGTTCAGATGTTCATGTTTGCTGGACATTATGA<br>CCTAGTTTTCCTGCATTGTGAGATTCATCTCTGTGATTCTCTTAATGAACAGTGC<br>CAGCCTTCTTGCTCAAGAAGTCAAGTCCGCAGTGAAGTACCGGCCATCGACCTA<br>GCCCGGGTTCTAGATTTGGGGCCCATCACTCGGAGAGGTGCACAGTCTCCCGGT<br>GTCATGAATGGAACCCCTAGCACTGCAGGGTTCCTGGTGGCCTGGCCTATGGTC<br>CTCCTGACTGTCCTCCTGGCTTGGCTGTTCTGA
