# Supplementary material for: Structure of human glycoprotein 2 reveals mechanisms underlying filament formation and adaption to proteolytic environment in the digestive tract
Source: PLoS Biol. 2025 Jun 23;23(6):e3003238. doi: 10.1371/journal.pbio.3003238 (PMC12212870; doi:10.1371/journal.pbio.3003238)
Supplement: S2 Table — (PDF) [file pbio.3003238.s017.pdf]

S2 Table Bands observed in western blotting shown in Figs 3a and S5a-d, and the plausible predictions of the residue range for each band

Band #	Positions in S5a-d Fig	Observed Mw. (kDa)	Predicted residue range	Mw. without glycans (kDa)	# of N-glycans	Contribution of each glycan† (kDa)
1	S5a Fig, 1st from top	105	28-524*	55.0	10	5
2	S5a Fig, 2nd from top<br>S5c Fig, 1st from top	70	185-524	37.9	7	4.6
3	S5c Fig, 2nd from top	62	185-456	30.5	7	4.5
4	S5c Fig, 3rd from top<br>S5d Fig, 1st from top	55	28-264	26.0	6	4.8
5	S5d Fig, 2nd from top	53	265-524	29.0	5	4.8
6	S5c Fig, 4th from top<br>S5d Fig, 3rd from top	45	28-218	21.1	5	4.8
7	S5b Fig, 1st from top<br>S5c Fig, 5th from top	36	185-339	17.3	4	4.7
8	S5b Fig, 2nd from top	34	340-524	20.5	3	4.5
9	S5c Fig, 6th form top	32	28-184	17.2	3	4.9
10	S5d Fig, 4th from top	28	28-184**	n.a.	n.a.	n.a.
11	S5b Fig, 3rd from top<br>S5c Fig, 7th from top<br>S5d Fig, 5th from top	27	340-456	13.3	3	4.6
12	S5a Fig, 3rd from top	25	28-184**	n.a.	n.a.	n.a.
13	S5b Fig, 4th from top	17	340-410	7.8	2	4.6
14	S5a Fig, 4th from top	15	28-184**	n.a.	n.a.	n.a.
15	S5d Fig, 6th from top	14	28-184**	n.a.	n.a.	n.a.
16	S5d Fig, 7th from top	13	265-339	8.4	1	4.6

†The estimated molecular weight displayed here was not used for the validation of N-glycans identified through mass spectrometry. *This residue range was validated by mass spectrometry; **The actual residue range should be smaller than this based on the observed molecular weight on the SDS-PAGE; Mw, molecular weight; n.a., not available.
