# Supplementary material for: Structure of human glycoprotein 2 reveals mechanisms underlying filament formation and adaption to proteolytic environment in the digestive tract
Source: PLoS Biol. 2025 Jun 23;23(6):e3003238. doi: 10.1371/journal.pbio.3003238 (PMC12212870; doi:10.1371/journal.pbio.3003238)
Supplement: S3 Table — (PDF) [file pbio.3003238.s018.pdf]

S3 Table N-Glycan types detected in this study when using GP2 sequence

Site	Structure	Glycan*	Peak area	Relative %	Protease	High-mannose
N122	(HexNAc)5(Hex)3(Fuc)1	1665.6	ND	ND	Trypsin	
N134	(HexNAc)2(Hex)3	910.3	3.59E+02	100	Chymotrypsin	Man3
	(HexNAc)4(Hex)5(Fuc)1	1786.7	ND	ND	Pepsin	
	(HexNAc)5(Hex)3(Fuc)1	1665.6	ND	ND	Chymotrypsin	
N204	(HexNAc)6(Hex)6(Fuc)3	2647.0	ND	ND	Trypsin	
N216	(HexNAc)8(Hex)8(Fuc)2	3231.2	ND	ND	Trypsin	
	(HexNAc)3(Hex)6	1599.6	ND	ND	Trypsin	
	**(HexNAc)8(Hex)9(Fuc)1**	3247.2	ND	ND (ND)	Trypsin	
N260	**(HexNAc)5(Hex)7(Fuc)1**	2313.8	3.48E+03	100% (100%)	Trypsin	
	(HexNAc)4(Hex)3	1316.5	ND	ND	Pepsin	
N291 (top 10)	**(HexNAc)3(Hex)6**	1599.6	3.23E+05	15.3% (12.2%)	Tryp./pep./chymo.	
	**(NexNAc)2(Hex)6**	1396.5	3.28E+05	15.0% (16.7%)	Tryp./pep./chymo.	Man6
	**(HexNAc)4(Hex)5(Fuc)2**	1932.7	2.33E+05	10.8% (9.5%)	Tryp./pep./chymo./ elastase	
	**(HexNAc)3(Hex)5**	1437.5	1.62E+05	7.5% (7.4%)	Tryp./pep./chymo.	
	**(HexNAc)2(Hex)5**	1234.4	1.41E+05	6.6% (5.1%)	Tryp./pep./chymo.	Man5
	**(HexNAc)4(Hex)4(Fuc)1**	1624.6	1.09E+05	5.1% (4.4%)	Tryp./pep./chymo.	
	**(HexNAc)3(Hex)5(Fuc)1**	1583.6	9.08E+04	4.2% (4.2%)	Tryp./chymo.	
	(HexNAc)3(Hex)4	1257.5	8.72E+04	4.1%	Tryp./pep./chymo.	
	(HexNAc)4(Hex)5	1640.6	8.35E+04	3.9%	Tryp./pep./chymo.	
	**(HexNAc)6(Hex)6(Fuc)3**	2647.0	7.46E+04	3.5% (4.8%)	Trypsin	
N342	(NexNAc)2(Hex)4	1072.4	1.64E+03	80.5%	Pepsin	Man4
	(HexNAc)2(Hex)5	1234.4	3.97E+02	19.5%	Chymotrypsin	Man5
N362	(NexNAc)4(Hex)5	1640.6	ND	ND	Pepsin	
N420	(NexNAc)4(Hex)3(Fuc)2	1608.6	1.01E+03	100%	Chymotrypsin	
	(NexNAc)4(Hex)5(Fuc)2	1932.7	ND	ND	Trypsin	

ND: not determined; *theoretical mass of the oligosaccharide was calculated using the online tool GlycanMass on Expasy (https://web.expasy.org/glycanmass). The glycan types validated using human proteome database are labeled in bold, with their relative percentage in the human proteome database search shown in parentheses. N-glycan types for which the glycopeptides containing them share a similar mass with those detected using the GP2-free database are labeled with grey background.
