# Supplementary material for: Structure of human glycoprotein 2 reveals mechanisms underlying filament formation and adaption to proteolytic environment in the digestive tract
Source: PLoS Biol. 2025 Jun 23;23(6):e3003238. doi: 10.1371/journal.pbio.3003238 (PMC12212870; doi:10.1371/journal.pbio.3003238)
Supplement: S1 Raw Images — (PDF) [file pbio.3003238.s019.pdf]

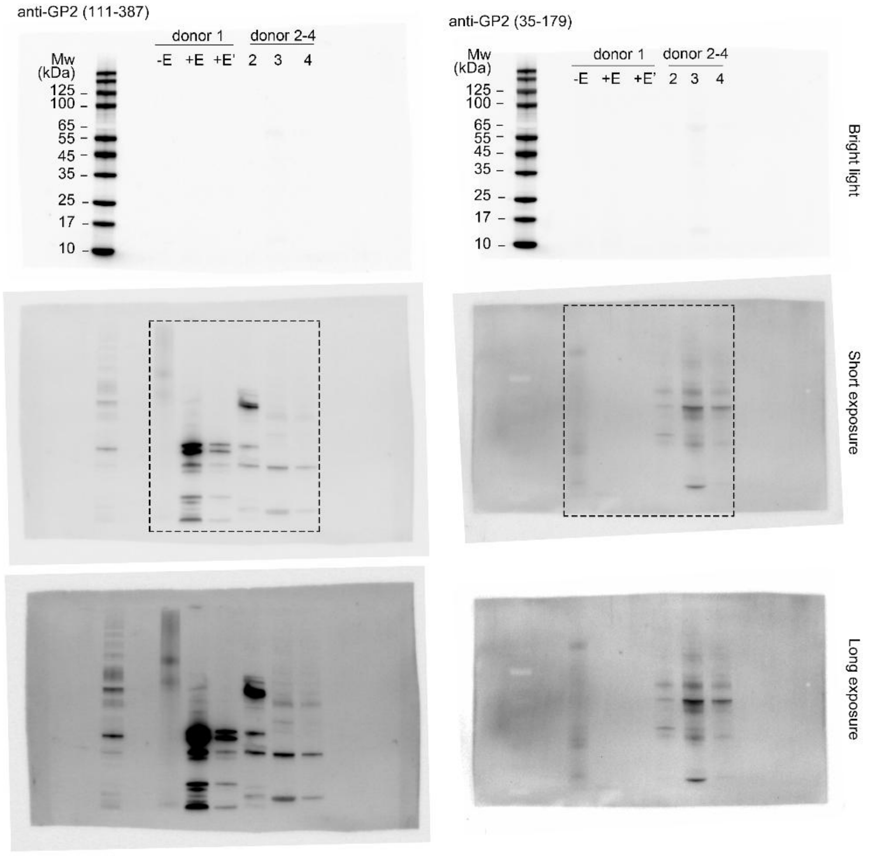

Uncropped version of blots shown in Fig 3a. Blots were visualized with bright light (upper panel) and chemiluminescence with short (middle panel) or long (bottom panel) exposures. -E, GP2 incubated without elastase; +E, GP2 incubated with elastase; +E', the same sample as +E, but the loading volume for SDS-PAGE was reduced to one-fifth; Mw, molecular weight. Dashed boxes represent the regions that were cropped and displayed in Fig 3a.

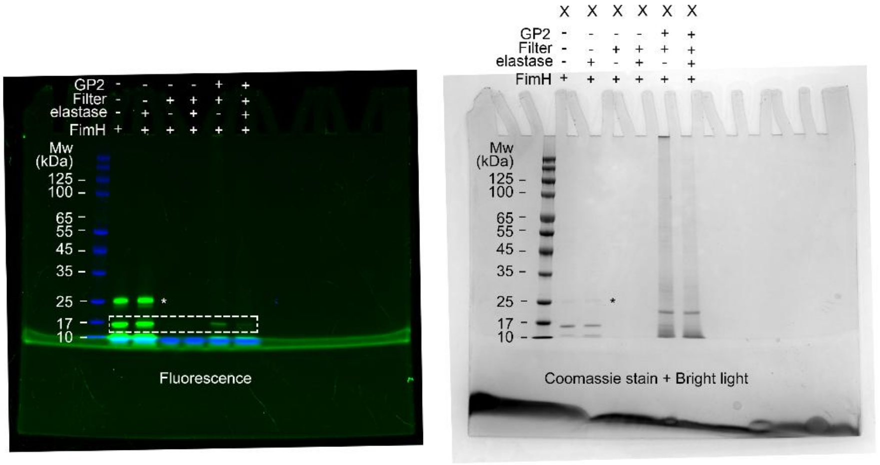

Uncropped version of gels shown in Fig 3c. The gels were visualized with fluorescence (left panel) and bright field (right panel). The band representing a contaminating protein during $FimH_L$ purification is labeled with an asterisk. Dashed boxes represent the regions that were

cropped, transferred to grey scale, and displayed in Fig 3c. The X labels indicate lanes that were labeled but not displayed in Fig 3c.

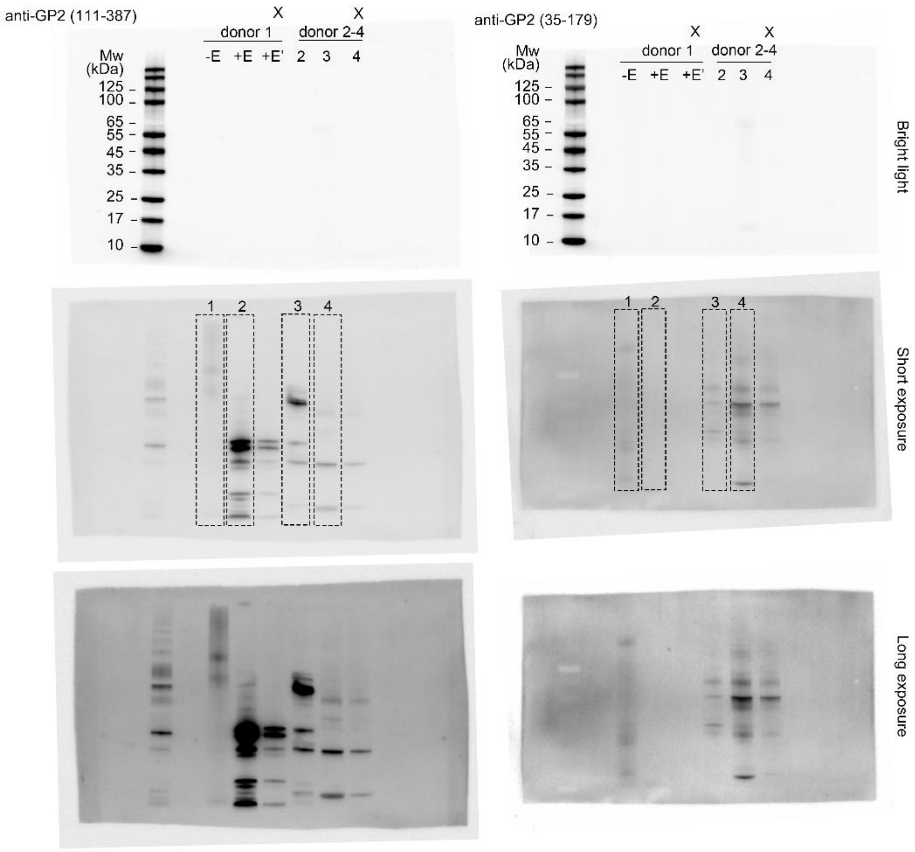

Uncropped version of blots shown in S6a-d Fig. These blots are the same as the original version of blots shown in Fig 3a, four lanes indicated with dashed boxes were cropped and shown in S6a-d Fig. The contrast of these four lanes were manually adjusted in S6 Fig for better presentation. The X labels indicate lanes that were labeled but not displayed in Fig 3c.

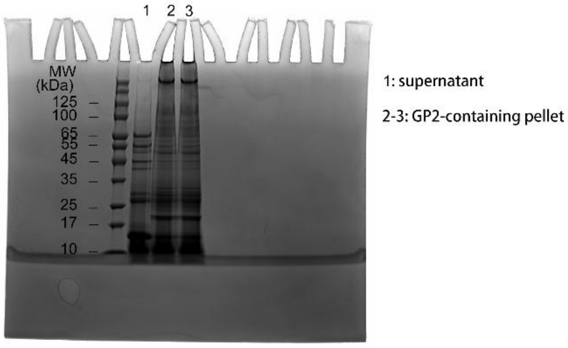

The original version of the gels shown in S7a Fig. The supernatant and pellet were fractions of the last centrifugation step in GP2 extraction.
