# Supplementary material for: Structure of human glycoprotein 2 reveals mechanisms underlying filament formation and adaption to proteolytic environment in the digestive tract
Source: PLoS Biol. 2025 Jun 23;23(6):e3003238. doi: 10.1371/journal.pbio.3003238 (PMC12212870; doi:10.1371/journal.pbio.3003238)
Supplement: S1 Supplementary Notes — (DOCX) [file pbio.3003238.s020.docx]

**Supplementary Notes 1-4**

***Supplementary Note 1***

The 105 kDa band (no. 1 in S2 Table), representing GP2 residues 28-524 as identified using mass spectrometry (see Methods), has a calculated molecular weight of 55 kDa without N-glycans. This suggests that the additional 50 kDa observed on SDS-PAGE should correspond to the presence of N-glycans. Mass spectrometry analysis identified nine N-glycosylation sites (S3 Table), and we also included a 10^th^ sites, Asn65, which was previously reported^1^ but was missed in this study. Due to the difficulty in predicting the exact influence of each N-glycan on the migration rate of SDS-PAGE, we made the assumption that all ten sites contribute similarly to the observed molecular weight on the gel, approximately 5 kDa per N-glycan site. This assumption served as a clue to predict the cleavage sites of the smaller bands observed in the GP2 samples.

We acknowledge that all predictions presented in the Supplementary Notes have not been validated with additional experiments, such as mass spectrometry. However, it is important to note that the main focus of the cleavage assays was to investigate whether the branch of GP2 could be cleaved from the filament core, rather than to identify specific cleavage site(s) in the filament core and branch. Therefore, in the Supplementary Notes, we provide plausible predictions regarding each smaller band based on the available clues, without further experimental validation.

The potential elastase cleavage sites on GP2 were predicted as follows (S5e-f Fig): The amino acid sequence within residue range of 181-480 was analyzed, and residues that formed a β-strand and cystine residues involved in di-sulfur bonds were colored black, while other residues were colored grey (S5e Fig). Subsequently, six types of residues (glycine, serine, alanine, valine, leucine, and isoleucine residues) that did not form a β-strand and were not directly adjacent to cystine residues involved in di-sulfur bonds were highlighted in yellow (S5e Fig). These residues were manually inspected in the cryo-EM structure of GP2, and those with side chains fully exposed to the solvent were further highlighted in red and considered as potential elastase cleavage sites (S5f Fig).

The 70 kDa band (no. 2 in S2 Table) is predicted to be the filament core of GP2 after cleavage at Ser184, based on the following observations: 1) this band is not visible with the branch antibody, suggesting that it is a C-terminal cleavage product of GP2; 2) the cleavage site Ser184 is one of the potential elastase cleavage site predicted above and it aligns with the reported elastase cleavage site Ser292 in UMOD^2^; 3) the calculated molecular weight, taking into account the remaining 7 N-glycosylation sites, matches well with the observed molecular weight on SDS-PAGE; 4) further inference suggests that the cleaved branch (containing residues 28-184) should have an molecular weight of 35 kDa (plus N-glycans), which is consistent with a previously reported recombinant decoy domain GP2^1^ (contains residues 8-181, approximately 35 kDa on SDS-PAGE). Considering this, the two bands at around 25 kDa and 15 kDa (no. 12 and no. 14 in S2 Table) likely represent more extensively cleaved products of GP2 branch, as they are smaller than 35 kDa and visible with the branch antibody.

***Supplementary Note 2***

Following elastase cleavage, we observed two prominent bands around 35 kDa that were exclusively detected with the filament core antibody (no. 7 and 8 in S2 Table), rather than the 70 kDa band before cleavage, which was predicted to represent the branch-free filament core of GP2 (Figs 3a, S5a-b). Given the comparable intensities of these two 35 kDa bands, it is reasonable to infer that they are the cleavage products derived from the 70 kDa band, with a cleavage site located in the middle of the filament core. Based on the predicted elastase cleavage sites, the most plausible cleavage site is residue 339, as this cleavage site would likely result in two fractions with similar observed molecular weights (no. 7 and 8 in S2 Table). Within the same sample, we also observed bands smaller than 35 kDa, which were only visible with the filament core antibody. These smaller bands are likely further cleavage products of the filament core. Although less certain, we have provided one potential set of residue range predictions for two more prominent bands (around 27 kDa, no.11 in S2 Table, and around 17 kDa, no. 13 in S2 Table). We hypothesize that these two bands represent cleavage products of the C-terminal half of the filament core, as the counterparts of these cleavages (e.g. peptide with residues 411-524 if the 17 kDa band contains residues 340-410) fall outside the range recognized by the filament core antibody, which explains why we did not observe them in the western blotting. Additionally, we observed a band at the migration front of the SDS-PAGE, and we have not provided a prediction for due to the difficulty in accurately estimating the molecular weight in this position.

We observed a difference in the amount of GP2 proteins on the western blotting between GP2 and eGP2, even though we loaded the same amount of (e)GP2 on the gel (Fig 3a). We believe it may be attributed to the stability of GP2 filaments. It is plausible that many GP2 filaments were resistant to denaturing by the SDS loading buffer, resulting in a smear pattern above 125 kDa (some may even be stacked at the loading well). By contrast, after elastase cleavage, the filaments may have become more susceptible to separation into polypeptides during SDS-PAGE sample preparation. Consequently, the protein amount on the western blotting may appear higher than in the sample before cleavage.

***Supplementary Note 3***

In the mass spectrometry data, we observed that the C-terminus of the detected regions corresponds to Trp524 (see Methods). Previous studies have indicated that the C-terminal transmembrane domain of GP2 (residues 518-537) is replaced by a glycosylphosphatidylinositol inositol (GPI) anchor in the Golgi apparatus^3^. Sequence analysis has suggested that Asn512 of GP2 is the GPI-anchor amidated asparagine^4^. These analyses imply that the sample used in the mass spectrometry study contains the ER from of GP2, which has not yet entered the Golgi apparatus. Considering the typical process of glycoprotein maturation involving high-mannose N-glycosylation in the ER and subsequent processing into hybrid- and complex-type glycans in the Golgi^5, 6^, the detection of the ER form of GP2 raises concerns regarding whether the glycan type detected in this study represent the mature form of GP2. However, based on the analysis of the N-glycosylation types identified using mass spectrometry, it is more likely that the main species in our sample is not immature ER from of GP2. Two reasons support this conclusion: i) it is known that the glycosylation in ER starts from Glc_3_Man_9_GlcNAc_2_ and usually ends with Man_8-9_GlcNAc_2_^5, 6^. Therefore, if the sample predominantly contained the ER form of GP2, all identified N-glycosylation sites should mainly exhibit these glycan types or their intermediates, but not hybrid- and complex-type, as they would not have undergone Golgi processing^5, 6^. However, Asn204 was determined to mainly carry hybrid-type glycan, Asn122 was identified to predominately contain complex-type glycans (S7 and S9 Figs, S3 Table), and hybrid- and complex-type glycans were detected in almost all glycosylation sites; ii) as described above, the majority of glycoproteins exiting the ER carry N-glycans with eight or nine mannose residues, with further mannose cleavage occurring in the Golgi^6^. In our mass spectrometry study, all detected high-mannose type glycans contained three to six mannose residues (S3 Table), suggesting they have undergone additional processing in the Golgi. Based on the analysis above, we believe it is reasonable to conclude that the identified N-glycosylation types in this study represents the mature form of GP2, despite the detectable contamination of the ER proteins.

***Supplementary Note 4***

Among all the bands visible with the branch antibody in samples from the small intestine, we believe that the 32 kDa band in donor 2 (no.9 in S2 Table) represents the fraction that is closest to the intact cleaved branch. Based on the previously mentioned predictions, the observed molecular weight of the intact branch (containing residues 28-184) should be around 35 kDa. Following this inference, the two bands that larger than 35 kDa in donor 2-4 (55 kDa, no. 4 in S2 Table, and 45 kDa, no.6 in S2 Table) should contain additional residues beyond 28-184. Considering the observed molecular weight and predicted cleavage sites, we propose that residues 28-264 and 28-218 are reasonable predictions for these two bands, respectively (S5c-d Fig). It is important to note that due to the presence of a di-sulfur bond between Cys211 of the branch and Cys241 of the filament core (S2g Fig), the branch containing residue Cys211 should be considered connected to the filament core. Therefore, the observation of these two larger bands would likely indicate that the branch was not fully cleaved from the GP2 filaments in these samples. Additionally, the two bands smaller than 35 kDa in donor 3-4 (28 kDa, no.10 in S2 Table, and 14 kDa, no. 15 in S2 Table) are likely representing more extensively cleaved fragments of the GP2 branch (S5d Fig).

Regarding the 62 kDa band in donor 2 (no. 3 in S2 Table), since it is only visible with the filament core antibody, similar to the 70 kDa band observed in pancreas-derived GP2, we believe it also represents the branch-free filament core (S5c Fig). However, compared to the 70 kDa band, the 62 kDa band may lack the C-terminal region due to protease cleavage. For the other bands that were visible with the filament core antibody in donor 2-4, we note that there are multiple possibilities, as we cannot confirm the cleavage sites solely based on the western blotting results. In S5 Fig and S2 Table, we have provided one set of plausible predictions of these bands, aiming to demonstrate that it is possible to maintain full coverage of the filament cores with these bands (with the C-terminal fractions not visible in both the branch antibody and filament core antibody). Therefore, these observations do not contradict our hypothesis that the GP2 filament core remains structurally intact in the human small intestines.

**Reference**

1. Stsiapanava A, Xu C, Nishio S, Han L, Yamakawa N, Carroni M, et al. Structure of the decoy module of human glycoprotein 2 and uromodulin and its interaction with bacterial adhesin FimH. *Nature Structural & Molecular Biology*.29(3),190-3(2022).

2. Weiss GL, Stanisich JJ, Sauer MM, Lin CW, Eras J, Zyla DS, et al. Architecture and function of human uromodulin filaments in urinary tract infections. *Science*.369(6506),1005-10(2020).

3. Zhang Z, Tanaka I, Nakahashi-Ouchida R, Ernst PB, Kiyono H, Kurashima Y. Glycoprotein 2 as a gut gate keeper for mucosal equilibrium between inflammation and immunity. *Seminars in Immunopathology*.45(4),493-507(2024).

4. UniProt: the Universal Protein Knowledgebase in 2023. *Nucleic Acids Res*.51(D1),D523-d31(2023).

5. Moremen KW, Tiemeyer M, Nairn AV. Vertebrate protein glycosylation: diversity, synthesis and function. *Nature Reviews Molecular Cell Biology*.13(7),448-62(2012).

6. Stanley P, Moremen KW, Lewis NE, Taniguchi N, Aebi M. N-Glycans. *Essentials of Glycobiology*.(Cold Spring Harbor Laboratory Press, Cold Spring Harbor (NY) 2022)
